# Serotypes in Adult Pneumococcal Pneumonia in Spain in the Era of Conjugate Vaccines

**DOI:** 10.3390/microorganisms9112245

**Published:** 2021-10-28

**Authors:** Lucía Fernández-Delgado, Jordi Càmara, Aida González-Díaz, Immaculada Grau, Hisashi Shoji, Fe Tubau, Sara Martí, Mª Ángeles Domínguez, Jordi Carratalà, Jose Yuste, Carmen Ardanuy

**Affiliations:** 1 Department of Microbiology, Hospital Universitari de Bellvitge-University of Barcelona-IDIBELL, 08907 Barcelona, Spain; lfernandezd@bellvitgehospital.cat (L.F.-D.); agonzalezd@bellvitgehospital.cat (A.G.-D.); hnkmtbcn@gmail.com (H.S.); f.tubau@bellvitgehospital.cat (F.T.); smartinm@bellvitgehospital.cat (S.M.); adominguez@bellvitgehospital.cat (M.Á.D.); 2 CIBER de Enfermedades Respiratorias (CIBERES), Instituto de Salud Carlos III, 28020 Madrid, Spain; icgrau@bellvitgehospital.cat (I.G.); jyuste@isciii.es (J.Y.); 3 Department of Infectious Diseases, Hospital Universitari de Bellvitge-University of Barcelona-IDIBELL, 08907 Barcelona, Spain; jcarratala@bellvitgehospital.cat; 4 Spanish Network for Research in Infectious Diseases (REIPI), Instituto de Salud Carlos III, 28029 Madrid, Spain; 5 Departament of Pathology and Experimental Therapeutics, University of Barcelona, 08007 Barcelona, Spain; 6 Pneumococcal Reference Laboratory, Centro Nacional de Microbiología, Instituto de Salud Carlos III, 28029 Madrid, Spain

**Keywords:** *Streptococcus pneumoniae*, pneumonia, serotype, antimicrobial resistance, pneumococcal conjugate vaccines (PCVs)

## Abstract

We studied changes in serotype distribution and antimicrobial susceptibility in adult pneumococcal pneumonia in Spain (2011–2019). Among 895 pneumococci collected (433 bacteremic [BPP] and 462 non-bacteremic [non-BPP]), serotypes 3 (17%), 19A (10%), 8 (6.7%) and 11A (6.7%) were the most frequent. Serotypes 16F, 19A and 24F were associated with old people (≥65) and serotypes 4, 7F, 8, 12F and 19F to young adults. Serotypes 12F, 24F and 1 were significantly more frequent in BPP and serotypes 11A, 23A and 19F in non-BPP. Amoxicillin resistance was higher in non-BPP (17% vs. 11%) while penicillin non-susceptibility (37% vs. 24%) and macrolide resistance (29% vs. 14%) were higher in older adults. In the period 2017–2019, the vaccine coverages were: 32% (PCV13), 39% (PCV15), 65% (PCV20) and 69% (PPV23). Differences were found in serotype composition and antimicrobial resistance by age and type of infection. The maintenance of serotype 3 as a leading cause of adult pneumococcal pneumonia and the increase in highly invasive (serotype 8) or antimicrobial-resistant (serotype 11A) serotypes is worrisome. Further studies will be required to analyse the impact of the upcoming broader conjugate vaccines.

## 1. Introduction

Lower respiratory tract infections are an important cause of severe disease worldwide. In children, *Streptococcus pneumoniae* is an important cause of invasive and non-invasive diseases [1]. Furthermore, it is among the leading causes of pneumonia in adults [2] with a significant impact on hospital admissions, morbidity/mortality and medical costs [3].

The capsule is the main pneumococcal virulence factor and also the basis for vaccine development. Vaccines are useful to prevent pneumococcal diseases, but the diversity of the capsular polysaccharide (at least 100 pneumococcal capsular types described) limits its effectiveness. The introduction of pneumococcal conjugate vaccines (PCVs) in children changed the epidemiology of pneumococcal diseases worldwide. In Spain, the current PCVs have been available on voluntary basis since 2009 (PCV10) and 2010 (PCV13) for children, and since 2012 (PCV13) for adults. PCV13 was included in the Spanish paediatric official vaccination schedule in 2016. The introduction of PCV13 in 2010 caused a sharp decrease in the incidence of invasive pneumococcal disease (IPD) in adults due to herd protection [4]. PCV13 also showed to be effective in preventing pneumococcal pneumonia caused by vaccine types [5]. Despite this, the impact of PCVs on the incidence of the different vaccine serotypes has not been homogeneous. For instance, no significant decrease in pneumococcal diseases caused by serotype 3 has been observed after the introduction of PCV13 [6].

Besides PCVs, the 23-valent pneumococcal polysaccharide vaccine (PPV23) is also included in the Spanish systematic vaccination calendar (adults over 65 years old) since 2004, with a vaccine uptake of 68.9% in 2017 [7]. As PPV23 does not prevent colonization, its impact on the epidemiology of the pneumococcal serotypes is limited. PPV23 has been shown to be effective in preventing IPD [8] but its efficacy in preventing pneumococcal pneumonia is controversial [9,10].

In this scenario, the serotype distribution of pneumococci causing IPD has been extensively analysed, especially in the PCVs era. However, data on pneumococcal serotypes causing pneumonia in adults, including bacteremic and non-bacteremic episodes, have been less studied. In this study, we aimed to describe the serotype distribution and antimicrobial susceptibility of pneumococci causing pneumonia in adults in Spain in the PCVs era. The data on the coverage of serotypes of current and upcoming vaccines should be useful to anticipate the evolution of pneumococcal diseases in the coming years.

## 2. Materials and Methods

### 2.1. Hospital Setting, Study Period and Definitions

This study focuses on the analysis of *Streptococcus pneumoniae* isolates collected from patients attended at Hospital Universitari de Bellvitge (HUB), located in a Southern Barcelona Area (Spain) with a diagnosis of pneumonia during 2011–2019. At HUB, all pneumococcal strains are prospectively collected and recorded in a database. The clinical charts of patients with *S. pneumoniae* isolation from both blood and respiratory specimens were retrospectively reviewed.

Pneumococcal pneumonia was defined as the isolation of *S. pneumoniae* from blood or respiratory samples in a patient with a chest X-ray compatible with pneumonia and signs and symptoms of infection. The isolation of pneumococci in blood in a patient with pneumonia was defined as bacteremic pneumococcal pneumonia (BPP). The isolation of *S. pneumoniae* from respiratory tract specimens was defined as non-bacteremic pneumonia (non-BPP). For sputum samples, only good quality sputum specimens (<10 squamous cells and >25 leukocytes per low-power field) were included. Pneumococcal pneumonia episodes with strain isolation only in the pleural fluid were included in non-BPP.

### 2.2. Bacterial Identification, Antimicrobial Susceptibility Testing and Serotyping

*S. pneumoniae* isolates were identified by standard microbiological procedures (optochin susceptibility and/or bile solubility). Antimicrobial susceptibility was performed through microdilution following the EUCAST (European Committee on Antimicrobial Susceptibility Testing) recommendations and criteria [11]. Serotype detection was performed by either a multiplex PCR protocol provided by the CDC [12] or the Quellung reaction at the Spanish Reference Laboratory for Pneumococci in Majadahonda, Madrid. For analysis purposes serotypes were classified in different schemes: PCV13 (1, 3, 4, 5, 6A, 6B, 7F, 9V, 14, 18C, 19A, 19F and 23F) and non-PCV13 (serotypes not included in PCV13); or PPV23 serotypes which includes serotypes 1, 2, 3, 4, 5, 6B, 7F, 8, 9N, 9V, 10A, 11A, 12F, 14, 15B, 17F, 18C, 19A, 19F, 20, 22F, 23F, and 33F; PCV15 (PCV13 serotypes plus 22F and 33F) and PCV20 (PCV13 plus serotypes 8, 10A, 11A, 12F, 15B, 22F and 33F).

According to the introduction of PCV13 for children, three periods were established: 2011–13 (early-PCV13), 2014–16 (late-PCV13) and 2017–19 (universal PCV13). For the early and late PCV13 periods, the paediatric vaccination occurred on a voluntary basis with a vaccine uptake of 78% [13].

### 2.3. Statistical Analysis

Statistical analyses were carried out using the SPSS software package (SPSS, version 18.0). Differences were assessed using the chi-square or Fisher’s exact tests, when appropriate. Two-sided *p* values < 0.05 were considered statistically significant.

## 3. Results

During the study period, a total of 895 pneumococcal strains were collected from patients with pneumococcal pneumonia. Of these, 580 occurred in men (64.8%) with a mean age of 67.6 (range 22–98). By age group, 124 episodes occurred in people aged 18–50, 212 in those aged 51–64, 224 in those aged 65–75 and 335 episodes were detected in patients older than 75. A total of 433 pneumococcal pneumonia episodes were obtained from blood, thus considered bacteremic (BPP, blood culture [*n* = 375], blood plus respiratory tract sample [*n* = 49], blood plus pleural fluid [*n* = 9]), and 462 were non-bacteremic (non-BPP, sputum [*n* = 409], bronchoalveolar lavage [*n* = 35] and pleural fluid [*n* = 18]).

### 3.1. Changes in Serotype Distribution over the Study Period

**Figure 1:**
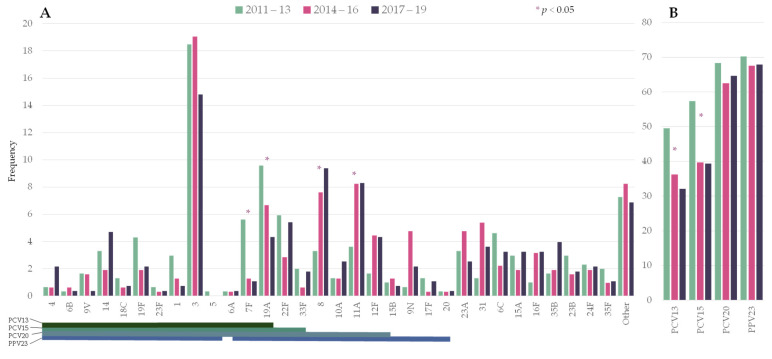
Changes in the serotype distribution of pneumococci causing pneumococcal pneumonia by period. (**A**) Individual serotypes; (**B**) Frequency of episodes caused by serotypes included in pneumococcal vaccines. Asterisks indicate significant changes between 2011–2013 and 2017–19. Lines below the serotypes indicate the serotype composition of the different vaccines. PCV13: 13-valent pneumococcal conjugate vaccine (PCV); PCV15: 15-valent PCV; PCV20: 20-valent PCV; PPV23: 23-valent pneumococcal polysaccharide.

The distribution of the number of episodes by period was: 303 in 2011–2013, 315 in 2014–2016, and 277 in 2017–2019. Figure 1 shows the frequency of the pneumococcal serotypes by period. Among PCV13 serotypes, serotypes 7F (5.6% vs. 1.1%) and 19A (9.6% vs. 4.3%) significantly decreased from 2011–2013 to 2017–2019. Serotype 3 ranked first in all three periods (18.5%, 19.0%, and 14.8%, respectively). Two non-PCV13 serotypes showed a statistically significant increase: serotypes 8 (3.3% vs. 9.4%) and 11A (3.6% vs. 8.3%).

Overall, PCV13 serotypes declined from 49.5% to 32.1% over the study period (RR 0.649 [0.5285–0.7971]). In the last period, the putative coverage for the upcoming conjugate vaccines PCV15 and PCV20 were 39% and 65%, respectively.

### 3.2. Distribution of Pneumococcal Serotypes Causing Pneumonia by Age Group

**Figure 2:**
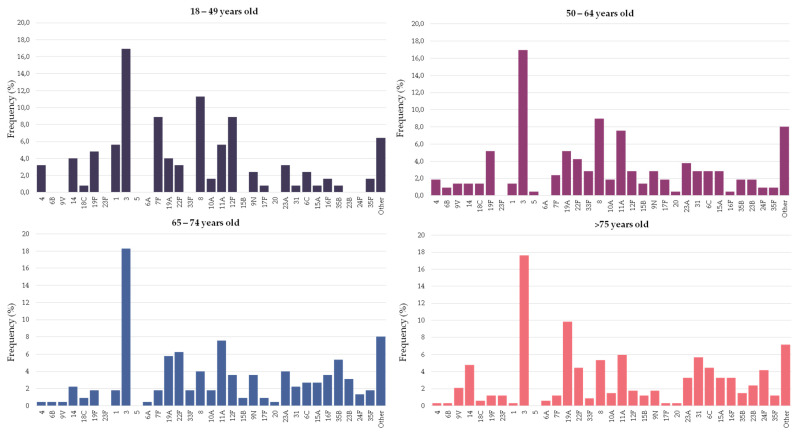
Serotype distribution of pneumococci causing pneumonia by age group. Bars indicate the frequency of each serotype in the particular age group.

Figure 2 and Table 1 show the serotype distribution by age group. Serotype 3 was the most frequent serotype in all age groups (range 17–18%). Serotypes 4, 7F, 8, 12F and 19F were statistically more represented in people younger than 65 years old and accounted for 27% of the episodes (11% for people ≥65 years old). Among these serotypes, serotype 8 alone accounted for 10% of the episodes in younger adults (5% in people ≥65 years old). On the other hand, serotypes 19A, 16F and 24F were more commonly found in older adults, accounting for 15% of the episodes (6% in younger adults). In the last period (2017–2019), the putative coverage of the different pneumococcal vaccines were higher in adults aged 18–64 years old than among people over 65: PCV13 (42% vs. 38%), PCV15 (48% vs. 44%), PCV20 (72% vs. 61%, *p* < 0.001) and PPV23 (77% vs. 64%, *p* < 0.001).

### 3.3. Differences in the Serotype Composition of Pneumococci Causing Bacteremic and Non-Bacteremic Pneumococcal Pneumonia

**Figure 3:**
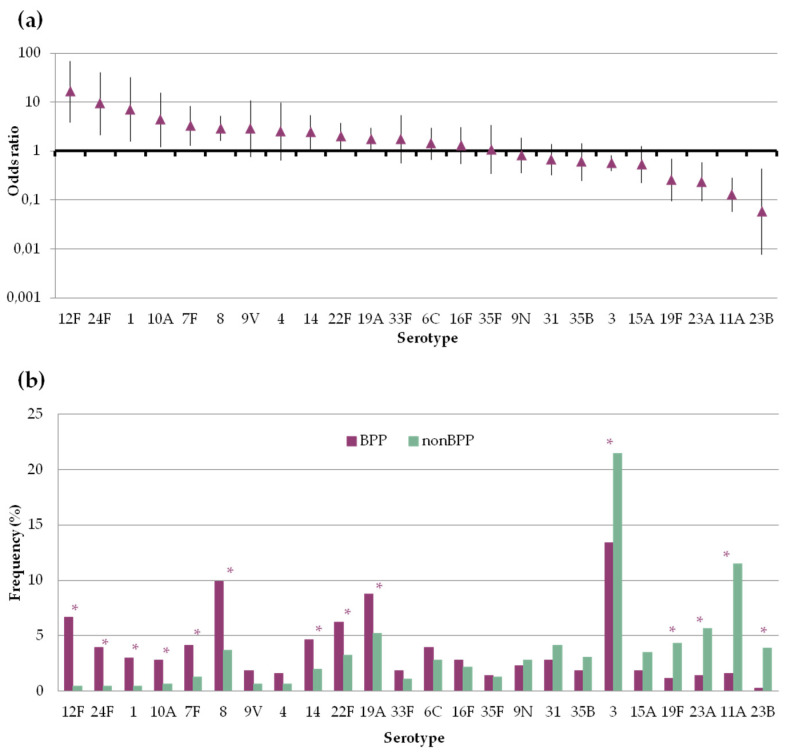
Frequency of pneumococcal serotypes in bacteremic (BPP) and non-bacteremic pneumonia (non-BPP). Only serotypes accounting for ≥10 episodes are shown (**a**) Serotype specific odds ratio and 95% CI comparing BPP and non-BPP. (**b**) Serotype distribution of pneumococci causing BPP or non-BPP. Asterisks indicate statistically significant changes (*p* < 0.05).

There was great variation in the propensity of the serotypes to be found in BPP or non-BPP episodes (Figure 3, Appendix A). Serotypes 1, 7F, 8, 10A, 12F, 14, 19A, 22F and 24F were significantly more frequent among BPP episodes, with ORs above 1. On the other hand, serotypes 3, 11A, 19F, 23A and 23B were more frequent among non-BPP episodes and had ORs below 1. In spite of that, serotypes 3 and 8 were the most frequent serotypes causing BPP, accounting for more than a fifth of episodes. With regard to non-BPP episodes, these were dominated by the occurrence of serotypes 3 and 11A.

### 3.4. Antimicrobial Susceptibility

We compared the antimicrobial non-susceptibility rates of pneumococci causing BPP and non-BPP. Antimicrobial resistance rates of non-BPP episodes were globally the highest (Table 2), although statistically significant differences were only found for amoxicillin (11% in BPP vs. 17% in non-BPP). By age group, pneumococci found in adults over 65 years old also showed the highest rates of resistance. These differences were statistically significant for penicillin (24% vs. 30%) and erythromycin (18% vs. 24%).

Through the study period, a reduction in the proportion of non-susceptible pneumococcal strains was observed for most antimicrobials. These differences were statistically significant for erythromycin (27% vs. 19%, 2011–2013 vs. 2017–2019). Furthermore, an increase in the MIC_90_ of penicillin and amoxicillin was observed (from 1 to 2 mg/L) and was associated with the increase in serotype 11A isolates.

## 4. Discussion

Lower respiratory tract infections are an important cause of morbidity and mortality worldwide, with *S. pneumoniae* being one of the main causative pathogens. Nevertheless, there is little available information on the serotype distribution and antimicrobial susceptibility of pneumococci isolated from bacteremic and non-bacteremic pneumococcal pneumonia after the introduction of PCVs.

In recent years, the introduction of PCVs has led to an overall decrease in the incidence of IPD, but an increase in disease caused by non-vaccine serotypes has also been detected [14]. In Spain, after the PCV13 introduction in 2010, there has been a significant decrease in the incidence of invasive infections caused by PCV13 serotypes, except for serotype 3 [15]. In our series, serotype 3 (included in PCV13 and PPV23) was the most frequent serotype among both BPP and non-BPP episodes. This is in agreement with recently published data showing that the burden of pneumonia due to PCV13 serotypes in Spain remains high, mainly due to serotype 3, which accounted for 66% of pneumonia episodes caused by vaccine types in the period 2017–2018 [16]. Similarly, serotype 3 was the leading cause of pneumococcal community-acquired pneumonia (CAP) in adults in the UK over the period 2016–2018 and the second most frequent serotype in adults that required hospitalization in the USA over 2013–2016 [17,18]. These results are probably related to the low efficacy of PCV13 to prevent serotype 3 colonization and disease in children which could limit the herd effect in adults. In this respect, a decrease in vaccine effectiveness of the six additional serotypes included in PCV13 compared to PCV7 has been described (73% vs. 90%) [6]. Additionally, serotype 3 pneumococci have been reported to release significant amounts of capsular polysaccharides during growth that may interfere with antibody-mediated killing [19]. It seems that the level of protection required to prevent serotype 3 diseases has not been achieved by current PCVs and this will require a different strategy.

The second serotype in frequency in our series was serotype 8, which also has been recently reported among the most frequent cause of IPD in adults in Spain and other European countries [14,20,21]. Serotype 8 was detected more frequently among young adults and bacteremic pneumonia, which is consistent with the high invasive disease potential described for this serotype [22]. The increased incidence of this serotype is not surprising, as it is not included in currently available PCVs. In our setting, serotype 8 is mostly associated with two main clonal complexes (CC53 and CC404) [15], both related to penicillin-susceptible isolates. The fact that this serotype was less frequent among pneumonia episodes in older adults could be associated with this antimicrobial susceptibility pattern, as it has been described that antimicrobial-resistant pneumococci appear more frequently in older patients with comorbidities [23]. Nevertheless, we did not analyse the putative contribution of PPV23 vaccination of older adults to the lower occurrence of this serotype in this age group. Either way, the introduction of the upcoming PCV20 should help to reduce the burden of pneumococcal diseases caused by serotype 8 in all age groups.

As observed for serotype 8, other invasive and usually antimicrobial susceptible serotypes such as 12F, 7F and 4 (all included in PPV23) appeared more frequently in young adults [24]. The exception to this pattern was serotype 19F which appeared more frequently in young adults but caused mostly non-BPP and was associated with penicillin non-susceptibility. Recent data on CAP episodes from different geographical areas show that serotype 19F is the second serotype in frequency among CAP episodes and is mostly associated with penicillin non-susceptibility [25]. An increase in the frequency of colonization by serotype 19F pneumococci has also been reported in Alaska which could be related to the introduction of a new genotype and subsequent reduced effectiveness of PCV7 [26]. In the USA, the number of serotype 19F isolates causing pneumococcal pneumonia in adults increased over the period 2009–2017 [27]. Although there is no clear explanation for the resurgence of serotype 19F isolates, it has been hypothesized that the high diversity of the capsular operon of serogroup 19 isolates could give them an evolutionary advantage to persist despite the introduction of PCVs [28]. In Spain, the incidence of serotype 19F isolates causing IPD decreased after the introduction of PCVs and is currently low [20]. In spite of that, its persistence among pneumococcal pneumonia episodes in young adults needs close surveillance.

Serotype 19A (included in PCV13) and serotypes 24F and 16F (both not included in any vaccine) were associated to appear more frequently in older adults. These data are concordant with previously published studies, showing a high propensity of serotype 16F to be found in the carriage and in persons with comorbidities [22,29]. Regarding serotype 19A, its incidence in IPD has decreased after PCV13 [15] and we observed the same decreasing trend in our study. Despite this, the rate of reduction in isolates of serotype 19A appeared to have been slower than that of additional PCV13 serotypes such as 1, 5 and 7F. As this serotype show a multidrug-resistant phenotype, [30], this could favour its persistence among adults with comorbidities. With regard to serotype 24F, it is a serotype whose incidence seems to be increasing [31], it is also associated with high invasive disease potential [32] and in our geographical area is related to the multidrug-resistant Denmark^14^-ST230 clone [23]. Probably this serotype has occupied the ecological niche that other multi-resistant serotypes such as 19A or 23F left in older adults after the introduction of PCVs. Furthermore, as this serotype is not included in the present or upcoming PCVs nor PPV23, people at risk of having IPD like older adults will not be protected. Then, a possible increase in the burden of serotype 24F disease should be monitored in the forthcoming years.

An interesting aspect of this study is the comparative analysis of the serotype distribution in BPP and non-BPP. In this way, serotype 3 was the most common serotype found in both types of episodes; however, it was highly prevalent in non-BPP cases. This fact emphasizes again the problem already observed concerning PCV13 effectiveness against this serotype. We also found a significantly high frequency of serotype 11A isolates causing non-BPP episodes. Studies on IPD show a low frequency of episodes caused by this serotype [33] but this is not the case when we look at other pneumococcal diseases. For instance, serotype 11A is a frequent serotype causing acute exacerbations and pneumonia in patients with chronic obstructive pulmonary disease (COPD) [34,35]. In fact, serotype 11A is also frequent in carriage suggesting a low invasive disease potential [36,37]. An increase in serotype 11A isolates has been reported in Europe after the introduction of PCVs [33] due to the emergence of a new lineage related to the Spain^9V^-156 clone (PMEN3). As this clone shows characteristically β-lactam resistance with high amoxicillin MICs (4–8 mg/L) [38], its significant frequency among non-BPP episodes should be taken into account when choosing empirical oral therapy. It will be interesting to monitor the impact of the upcoming PCV20 on serotype 11A which has a high carriage frequency.

We also explored differences in antimicrobial susceptibility between age groups and between BPP and non-BPP episodes. In general, higher percentages of non-susceptibility were observed for those non-BPP episodes and those that occurred in people over 65 years old. Statistically significant differences were evidenced for amoxicillin, which showed a higher percentage of non-susceptibility in non-BPP episodes, and penicillin and erythromycin which had a higher frequency of non-susceptibility in the elderly. As described before, the frequent occurrence of 11A isolates with high amoxicillin MICs in non-BPP could justify these differences. On the other hand, since antimicrobial resistance is usually a consequence of antimicrobial consumption [39], the existence of a higher proportion of isolates non-susceptible to penicillin and erythromycin in the elderly could be a consequence of the frequent use of β-lactam and macrolide antibiotics in patients with chronic conditions such as COPD. Therefore, empirical therapy should be carefully chosen in those patients with a history of antibiotic use.

Our study has some limitations that should be acknowledged. First, the data are local and could not be extrapolated to other settings. Second, we have no data regarding the vaccination status of the patients which could influence the prevalence of certain serotypes. In the same way, we have no data regarding comorbidities that could have increased over the study period changing the epidemiology and incidence of pneumococcal diseases. Nevertheless, our study includes a long period of time and a significant number of bacteremic and non-bacteremic episodes which is among the main strengths of our study. It should be noted that collecting good quality sputum is difficult and it is not routinely collected in some hospitals. Since not all episodes of pneumococcal pneumonia led to invasive disease, it is mandatory to also study the population of pneumococci collected from non-invasive specimens.

In summary, our study shows a reduction in most PCV13 serotypes in adult pneumococcal pneumonia except serotype 3, suggesting low effectiveness of PCV13 to prevent serotype 3 diseases. The increase in certain non-PCV13 serotypes such as serotype 8 and 12F raises concern due to its invasiveness, but it is hoped that this will be balanced with the release of the next PCVs. With regard to non-BPP, the high frequency of serotype 11A isolates with amoxicillin resistance should be taken into account when choosing empiric antimicrobial therapy. Finally, the increase in serotype 24F, not included in the upcoming vaccines, deserves further surveillance. On account of all this, it is critical to monitor the evolution of pneumococcal diseases and the impact of the introduction of the upcoming conjugate vaccines.

## Figures and Tables

**Table 1:** Pneumococcal serotypes causing pneumonia in young and older adults. Data show the frequency of each serotype in the age group. Only serotypes accounting for ≥10 episodes are shown. Statistically significant results are highlighted in bold. NS: not statistically significant.

Serotype	Vaccine Type				18–64 (%) *n* = 336	>64 (%) *n* = 559	*p*-Value
	PCV13	PCV15	PCV20	PPV23			
3	X	X	X	X	17.0	17.9	NS
**8**			**X**	**X**	**9.8**	**4.8**	**0.006**
11A			X	X	6.8	6.6	NS
**12F**			**X**	**X**	**5.1**	**2.5**	**0.043**
**19F**	**X**	**X**	**X**	**X**	**5.1**	**1.4**	**0.002**
**19A**	**X**	**X**	**X**	**X**	**4.8**	**8.2**	**0.048**
**7F**	**X**	**X**	**X**	**X**	**4.8**	**1.4**	**0.005**
22F		X	X	X	3.9	5.2	NS
23A					3.6	3.6	NS
1	X	X	X	X	3.0	0.9	NS
9N				X	2.7	3.8	NS
6C					2.7	2.5	NS
14	X	X	X	X	2.4	3.8	NS
**4**	**X**	**X**	**X**	**X**	**2.4**	**0.4**	**0.014**
31					2.1	4.3	NS
15A					2.1	3.0	NS
10A			X	X	1.8	1.6	NS
33F		X	X	X	1.8	1.3	NS
35B					1.5	3.0	NS
23B					1.2	2.7	NS
35F					1.19	1.43	NS
**16F**					**0.9**	**3.4**	**0.033**
9V	X	X	X	X	0.9	3.0	NS
**24F**					**0.6**	**3.0**	**0.026**
Other					12.2	11.8	NS

**Table 2:** Changes in antimicrobial non-susceptibility rates by type of infection (**a**), age group (**b**) and period (**c**). Percentages correspond to EUCAST clinical breakpoints of non-susceptibility for penicillin (>0.06 mg/L), amoxicillin (>0.5 mg/L) and cefotaxime (>0.5 mg/L) and resistance for erythromycin (>0.5 mg/L) and levofloxacin (>2 mg/L). Statistically significant results are highlighted in bold. MIC_50_ and MIC_90_ are given in mg/L.

(**a**)	**BPP (*n* = 433)**			**Non-BPP (*n* = 462)**			
**Antibiotic**	**MIC_50_**	**MIC_90_**	**%**	**MIC_50_**	**MIC_90_**	**%**	***p*-Value**
Penicillin Amoxicillin	<0.06	1	26%	<0.06	2	29%	0.29
	<0.06	1	11%	<0.06	2	17%	**0.009**
Cefotaxime Erythromycin	<0.06	0.5	9%	<0.06	1	10%	0.63
	<0.25	>32	21%	<0.25	>32	23%	0.59
Levofloxacin	1	1	1%	0.5	1	2%	0.44
(**b**)	**18–64 (*n* = 336)**			**>65 (*n* = 559)**			
**Antibiotic**	**MIC_50_**	**MIC_90_**	**%**	**MIC_50_**	**MIC_90_**	**%**	***p*-Value**
Penicillin Amoxicillin	<0.06	1	24%	<0.06	2	30%	**0.045**
	<0.06	1	13%	<0.06	2	14%	0.76
Cefotaxime Erythromycin	<0.06	0.5	9%	<0.06	1	10%	0.48
	<0.25	>32	18%	<0.25	>32	24%	**0.037**
Levofloxacin	1	1	1%	1	1	2%	0.39
(**c**)	**2011–13 (*n* = 303)**			**2017–19 (*n* = 277)**			
**Antibiotic**	**MIC_50_**	**MIC_90_**	**%**	**MIC_50_**	**MIC_90_**	**%**	***p*-Value**
Penicillin Amoxicillin	<0.06	1	31%	<0.06	2	26%	0.15
	<0.06	1	14%	<0.06	2	15%	0.72
Cefotaxime Erythromycin	<0.06	0.5	10%	<0.06	0.5	8%	0.46
	<0.25	>32	27%	<0.25	>32	19%	**0.04**
Levofloxacin	1	1	2%	1	1	1%	0.7

## Data Availability

Data from this study is available in the figures and tables over the paper.

## Appendix A

**Table A1:** Distribution of pneumococcal serotypes causing bacteremic (BPP) and non-bacteremic pneumonia (non-BPP). The serotype-specific odds ratio and 95% CI comparing BPP and non-BPP are shown. Statistically significant results are highlighted in bold.

Serotype	BPP *n* = 433	Non–BPP *n* = 462	OR (95% CI)
12F	29	2	**16.51 (3.92–69.62)**
24F	17	2	**9.4 (2.16–40.93)**
1	13	2	**7.12 (1.6–31.73)**
10A	12	3	**4.36 (1.22–15.56)**
7F	18	6	**3.3 (1.3–8.38)**
8	43	17	**2.89 (1.62–5.14)**
9V	8	3	2.88 (0.76–10.93)
4	7	3	2.51 (0.65–9.78)
14	20	9	**2.44 (1.1–5.41)**
22F	27	15	**1.98 (1.04–3.78)**
19A	38	24	**1.76 (1.03–2.98)**
33F	8	5	1.72 (0.56–5.3)
6C	17	13	1.41 (0.68–2.94)
16F	12	10	1.29 (0.55–3.01)
35F	6	6	1.07 (0.34–3.34)
9N	10	13	0.82 (0.35–1.88)
31	12	19	0.66 (0.32–1.39)
35B	8	14	0.6 (0.25–1.45)
3	58	99	**0.57 (0.4–0.81)**
15A	8	16	0.52 (0.22–1.24)
19F	5	20	**0.26 (0.1–0.69)**
23A	6	26	**0.24 (0.1–0.58)**
11A	7	53	**0.13 (0.06–0.28)**
23B	1	18	**0.06 (0.01–0.43)**

## References

[1] Troeger C., Blacker B., Khalil I.A., Rao P.C., Cao J., Zimsen S.R.M., Albertson S.B., Deshpande A., Farag T., Abebe Z. (2018). Estimates of the global, regional, and national morbidity, mortality, and aetiologies of lower respiratory infections in 195 countries, 1990–2016: A systematic analysis for the Global Burden of Disease Study 2016. Lancet Infect. Dis..

[2] Simonetti A.F., van Werkhoven C.H., Schweitzer V.A., Viasus D., Carratalà J., Postma D.F., Oosterheert J.J., Bonten M.J.M. (2017). Predictors for individual patient antibiotic treatment effect in hospitalized community-acquired pneumonia patients. Clin. Microbiol. Infect..

[3] Darbà J., Marsà A. (2021). Hospital incidence, in-hospital mortality and medical costs of pneumococcal disease in Spain (2008–2017): A retrospective multicentre study. Curr. Med. Res. Opin..

[4] Càmara J., Marimón J.M., Cercenado E., Larrosa N., Quesada M.D., Fontanals D., Cubero M., Pérez-Trallero E., Fenoll A., Liñares J. (2017). Decrease of invasive pneumococcal disease (IPD) in adults after introduction of pneumococcal 13-valent conjugate vaccine in Spain. PLoS ONE.

[5] Bonten M.J.M., Huijts S.M., Bolkenbaas M., Webber C., Patterson S., Gault S., van Werkhoven C.H., van Deursen A.M.M., Sanders E.A.M., Verheij T.J.M. (2015). Polysaccharide Conjugate Vaccine against Pneumococcal Pneumonia in Adults. N. Engl. J. Med..

[6] Andrews N.J., Waight P.A., Burbidge P., Pearce E., Roalfe L., Zancolli M., Slack M., Ladhani S.N., Miller E., Goldblatt D. (2014). Serotype-specific effectiveness and correlates of protection for the 13-valent pneumococcal conjugate vaccine: A postlicensure indirect cohort study. Lancet Infect. Dis..

[7] Vila-Córcoles A., Ochoa-Gondar O., de Diego C., Satué E., Vila-Rovira A., Aragón M. (2019). Pneumococcal vaccination coverages by age, sex and specific underlying risk conditions among middle-aged and older adults in Catalonia, Spain, 2017. Eurosurveillance.

[8] Shapiro E.D., Berg A.T., Austrian R., Schroeder D., Parcells V., Margolis A., Adair R.K., Clemens J.D. (1991). The protective efficacy of polyvalent pneumococcal polysaccharide vaccine. N. Engl. J. Med..

[9] Koivula I., Stén M., Leinonen M., Mäkelä P.H. (1997). Clinical efficacy of pneumococcal vaccine in the elderly: A randomized, single-blind population-based trial. Am. J. Med..

[10] Ortqvist A., Hedlund J., Burman L.A., Elbel E., Hofer M., Leinonen M., Lindblad I., Sundelof S., Kalin M., Aufwerber E. (1998). Randomised trial of 23-valent pneumococcal capsular polysaccharide vaccine in prevention of pneumonia in middle-aged and elderly people. Lancet.

[11] The European Committee on Antimicrobial Susceptibility Testing (2021). Breakpoint Tables for Interpretation of MICs and Zone Diameters.

[12] (2006). List of Oligonucleotide Primers Used in 41 Conventional Multiplex PCR Assays for Pneumococcal Serotype Deduction of 70 Serotypes. CDC.

[13] Hanquet G., Krizova P., Valentiner-Branth P., Ladhani S.N., Nuorti J.P., Lepoutre A., Mereckiene J., Knol M., Winje B.A., Ciruela P. (2019). Effect of childhood pneumococcal conjugate vaccination on invasive disease in older adults of 10 European countries: Implications for adult vaccination. Thorax.

[14] Ladhani S.N., Collins S., Djennad A., Sheppard C.L., Borrow R., Fry N.K., Andrews N.J., Miller E., Ramsay M.E. (2018). Rapid increase in non-vaccine serotypes causing invasive pneumococcal disease in England and Wales, 2000–17: A prospective national observational cohort study. Lancet Infect. Dis..

[15] González-Díaz A., Càmara J., Ercibengoa M., Cercenado E., Larrosa N., Quesada M.D., Fontanals D., Cubero M., Marimón J.M., Yuste J. (2020). Emerging non-13-valent pneumococcal conjugate vaccine (PCV13) serotypes causing adult invasive pneumococcal disease in the late-PCV13 period in Spain. Clin. Microbiol. Infect..

[16] Torres A., Menéndez R., España P.P., Fernández-Villar J.A., Marimón J.M., Cilloniz C., Méndez R., Egurrola M., Botana-Rial M., Ercibengoa M. (2021). The Evolution and Distribution of Pneumococcal Serotypes in Adults Hospitalized With Community-Acquired Pneumonia in Spain Using a Serotype-Specific Urinary Antigen Detection Test: The CAPA Study, 2011–2018. Clin. Infect. Dis..

[17] Pick H., Daniel P., Rodrigo C., Bewick T., Ashton D., Lawrence H., Baskaran V., Edwards-Pritchard R.C., Sheppard C., Eletu S.D. (2020). Pneumococcal serotype trends, surveillance and risk factors in UK adult pneumonia, 2013-18. Thorax.

[18] Isturiz R., Grant L., Gray S., Alexander-Parrish R., Jiang Q., Jodar L., Peyrani P., Ford K.D., Pride M.W., Self W.H. (2021). Expanded Analysis of 20 Pneumococcal Serotypes Associated With Radiographically Confirmed Community-acquired Pneumonia in Hospitalized US Adults. Clin. Infect. Dis..

[19] Choi E.H., Zhang F., Lu Y., Malley R. (2016). Strains Reduces the Protective Effect of Anti-Type 3 CPS Antibodies. Clin. Vaccine Immunol..

[20] de Miguel S., Domenech M., González-Camacho F., Sempere J., Vicioso D., Sanz J.C., Comas L.G., Ardanuy C., Fenoll A., Yuste J. (2020). Nationwide Trends of Invasive Pneumococcal Disease in Spain From 2009 Through 2019 in Children and Adults During the Pneumococcal Conjugate Vaccine Era. Clin. Infect. Dis..

[21] Silva-Costa C., Gomes-Silva J., Teodoro I., Ramirez M., Melo-Cristino J. (2021). Invasive pneumococcal disease in adults in portugal: The importance of serotypes 8 and 3 (2015–2018). Microorganisms.

[22] Sá-Leao R., Pinto F., Aguiar S., Nunes S., Carriço J.A., Frazao N., Gonçalves-Sousa N., Melo-Cristino J., De Lencastre H., Ramirez M. (2011). Analysis of invasiveness of pneumococcal serotypes and clones circulating in portugal before widespread use of conjugate vaccines reveals heterogeneous behavior of clones expressing the same serotype. J. Clin. Microbiol..

[23] Càmara J., Grau I., González-Diáz A., Tubau F., Calatayud L., Cubero M., Domínguez M.Á., Linãres J., Yuste J., Pallarés R. (2021). A historical perspective of MDR invasive pneumococcal disease in Spanish adults. J. Antimicrob. Chemother..

[24] Lindstrand A., Galanis I., Darenberg J., Morfeldt E., Naucler P., Blennow M., Alfvén T., Henriques-Normark B., Örtqvist Å. (2016). Unaltered pneumococcal carriage prevalence due to expansion of non-vaccine types of low invasive potential 8 years after vaccine introduction in Stockholm, Sweden. Vaccine.

[25] McCurdy S.P., Sheets A.J., Cammarata S.K., Vidal J.E. (2021). Serotype distribution and antimicrobial susceptibility of Streptococcus pneumoniae isolates from a Phase III community-acquired bacterial pneumonia (CABP) trial. JAC-Antimicrob. Resist..

[26] Gounder P.P., Bruden D., Rudolph K., Zulz T., Hurlburt D., Thompson G., Bruce M.G., Hennessy T.W. (2018). Re-emergence of pneumococcal colonization by vaccine serotype 19F in persons aged ≥5 years after 13-valent pneumococcal conjugate vaccine introduction—Alaska, 2008–2013. Vaccine.

[27] Suaya J.A., Mendes R.E., Sings H.L., Arguedas A., Reinert R.R., Jodar L., Isturiz R.E., Gessner B.D. (2020). Streptococcus pneumoniae serotype distribution and antimicrobial nonsusceptibility trends among adults with pneumonia in the United States, 2009‒2017. J. Infect..

[28] Rockett R.J., Oftadeh S., Bachmann N.L., Timms V.J., Kong F., Gilbert G.L., Sintchenko V. (2018). Genome-wide analysis of Streptococcus pneumoniae serogroup 19 in the decade after the introduction of pneumococcal conjugate vaccines in Australia. Sci. Rep..

[29] Naucler P., Galanis I., Petropoulos A., Granath F., Morfeldt E., Örtqvist Å., Henriques-Normark B. (2021). Chronic disease and immunosuppression increase the risk for non-vaccine serotype pneumococcal disease—A nationwide population-based study. Clin. Infect. Dis..

[30] Muñoz-Almagro C., Esteva C., Fernandez de Sevilla M., Selva L., Gene A., Pallares R. (2009). Emergence of invasive pneumococcal disease caused by multidrug-resistant serotype 19A among children in Barcelona. J. Infect..

[31] Ouldali N., Varon E., Levy C., Angoulvant F., Georges S., Ploy M.C., Kempf M., Cremniter J., Cohen R., Bruhl D.L. (2021). Invasive pneumococcal disease incidence in children and adults in France during the pneumococcal conjugate vaccine era: An interrupted time-series analysis of data from a 17-year national prospective surveillance study. Lancet Infect. Dis..

[32] Phillips M.T., Warren J.L., Givon-Lavi N., Tothpal A., Regev-Yochay G., Dagan R., Weinberger D.M. (2020). Evaluating post-vaccine expansion patterns of pneumococcal serotypes. Vaccine.

[33] González-Díaz A., Machado M.P., Càmara J., Yuste J., Varon E., Domenech M., Del Grosso M., Marimón J.M., Cercenado E., Larrosa N. (2020). Two multi-fragment recombination events resulted in the β-lactam-resistant serotype 11A-ST6521 related to Spain9V-ST156 pneumococcal clone spreading in south-western Europe, 2008 to 2016. Eurosurveillance.

[34] Shoji H., Vázquez-Sánchez D.A., Gonzalez-Diaz A., Cubero M., Tubau F., Santos S., García-Somoza D., Liñares J., Yuste J., Martí S. (2018). Overview of pneumococcal serotypes and genotypes causing diseases in patients with chronic obstructive pulmonary disease in a Spanish hospital between 2013 and 2016. Infect. Drug Resist..

[35] Domenech A., Ardanuy C., Calatayud L., Santos S., Tubau F., Grau I., Verdaguer R., Dorca J., Pallares R., Martin R. (2011). Serotypes and genotypes of Streptococcus pneumoniae causing pneumonia and acute exacerbations in patients with chronic obstructive pulmonary disease. J. Antimicrob. Chemother..

[36] Van Hoek A.J., Sheppard C.L., Andrews N.J., Waight P.A., Slack M.P.E., Harrison T.G., Ladhani S.N., Miller E. (2014). Pneumococcal carriage in children and adults two years after introduction of the thirteen valent pneumococcal conjugate vaccine in England. Vaccine.

[37] Desmet S., Wouters I., Van Heirstraeten L., Beutels P., Van Damme P., Malhotra-Kumar S., Maes P., Verhaegen J., Peetermans W.E., Lagrou K. (2021). In-depth analysis of pneumococcal serotypes in Belgian children (2015–2018): Diversity, invasive disease potential, and antimicrobial susceptibility in carriage and disease. Vaccine.

[38] Càmara J., Cubero M., Martín-Galiano A.J., García E., Grau I., Nielsen J.B., Worning P., Tubau F., Pallarés R., Ángeles Domínguez M. (2018). Evolution of the b-lactam-resistant Streptococcus pneumoniae PMEN3 clone over a 30 year period in Barcelona, Spain. J. Antimicrob. Chemother..

[39] Riedel S., Beekmann S.E., Heilmann K.P., Richter S.S., Garcia-De-Lomas J., Ferech M., Goosens H., Doern G.V. (2007). Antimicrobial use in Europe and antimicrobial resistance in Streptococcus pneumoniae. Eur. J. Clin. Microbiol. Infect. Dis..

